# Impact of COVID-19 Pandemic on Patients with ST-Segment-Elevation Myocardial Infarction Complicated by Out-of-Hospital Cardiac Arrest

**DOI:** 10.3390/ijerph20010337

**Published:** 2022-12-26

**Authors:** Tomasz Tokarek, Artur Dziewierz, Aleksander Zeliaś, Krzysztof Piotr Malinowski, Tomasz Rakowski, Dariusz Dudek, Zbigniew Siudak

**Affiliations:** 1 Center for Invasive Cardiology, Electrotherapy and Angiology, 33-300 Nowy Sacz, Poland; 2 Center for Innovative Medical Education, Jagiellonian University Medical College, 30-688 Krakow, Poland; 3 Clinical Department of Cardiology and Cardiovascular Interventions, University Hospital, 30-688 Krakow, Poland; 4 2nd Department of Cardiology, Institute of Cardiology, Jagiellonian University Medical College, 30-688 Krakow, Poland; 5 Digital Medicine & Robotics Center, Jagiellonian University Medical College, 31-034 Krakow, Poland; 6 Faculty of Medicine and Health Science, Jan Kochanowski University, 25-317 Kielce, Poland

**Keywords:** STEMI, COVID-19, out-of-hospital cardiac arrest

## Abstract

Patients with ST-segment-elevation myocardial infarction (STEMI) treated during the COVID-19 pandemic might experience prolonged time to reperfusion. The delayed reperfusion may potentially aggravate the risk of out-of-hospital cardiac arrest (OHCA) in those patients. Limited access to healthcare, more reluctant health-seeking behaviors, and bystander readiness to render life-saving interventions might additionally contribute to the suggested change in the risk of OHCA in STEMI. Thus, we sought to explore the effects of the COVID-19 outbreak on treatment delay and clinical outcomes of patients with STEMI with OHCA. Overall, 5,501 consecutive patients with STEMI complicated by OHCA and treated with primary percutaneous coronary intervention with stent implantation were enrolled. A propensity score matching was used to obviate the possible impact of non-randomized design. A total of 740 matched pairs of patients with STEMI and OHCA treated before and during the COVID-19 pandemic were compared. A similar mortality and prevalence of periprocedural complications were observed in both groups. However, patients treated during the COVID-19 outbreak experienced longer delays from first medical contact to angiography (88.8 (±61.5) vs. 101.4 (±109.8) [minutes]; *p* = 0.006). There was also a trend toward prolonged time from pain onset to angiography in patients admitted to the hospital in the pandemic era (207.3 (±192.8) vs. 227.9 (±231.4) [minutes]; *p* = 0.06). In conclusion, the periprocedural outcomes in STEMI complicated by OHCA were comparable before and during the COVID-19 era. However, treatment in the COVID-19 outbreak was associated with a longer time from first medical contact to reperfusion.

## 1. Introduction

The COVID-19 pandemic engenders profound changes in the functioning and effectiveness of the healthcare system [1,2,3,4]. A sudden surge of infected patients and mandatory swab tests with pandemic-specific protocols forced fragmentation of attention and resources of medical staff. Thus, patients with ST-segment-elevation myocardial infarction (STEMI) might experience delays in treatment and prolonged time to achieve reperfusion. More importantly, delayed reperfusion may aggravate the risk of out-of-hospital cardiac arrest (OHCA) in those patients [2,3,4,5,6]. Furthermore, fear of contamination of SARS-CoV-2 might result in more reserved attitudes toward resuscitation practice and a lower rate of successful cardiopulmonary resuscitation (CPR) [7,8,9,10]. The population at risk remained constant; however, recent studies reported a growing incidence of OHCA and a decreased number of STEMI with detrimental outcomes during the pandemic era [7,8,9,10,11]. Limited access to healthcare, more reluctant health-seeking behaviors, and bystander readiness to render life-saving interventions might additionally contribute to the suggested change in the risk of OHCA in STEMI [4,5,6,7,8,9,10,11]. Despite the remarkable growth of evidence, there is a lack of data on clinical outcomes in STEMI complicated by OHCA during the COVID-19 pandemic compared to the pre-pandemic era. Since randomized comparison is unfeasible, only all-comers studies might shed light on this topic. Thus, we sought to explore the effects of the COVID-19 outbreak on treatment delay and clinical outcomes of patients with STEMI with OHCA treated with percutaneous coronary intervention (PCI).

## 2. Materials and Methods

**Figure 1:**
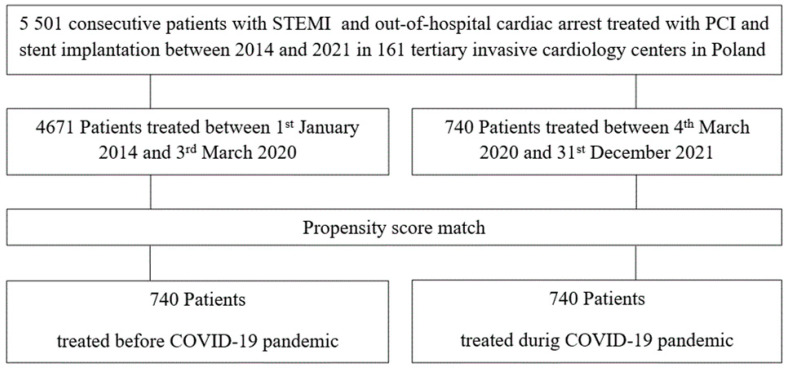
Flow chart of included patients. PCI—percutaneous coronary intervention; STEMI—ST-segment-elevation myocardial.

A comprehensive elucidation of the ORPKI national PCI registry was described in former studies [3,12,13,14,15,16]. In brief, ORPKI is an electronic database collecting data on all PCI procedures in interventional cardiology carried out in Poland. Endorsement for this registry was provided by the Association of Cardiovascular Interventions of the Polish Cardiac Society [5,17]. Currently, it is managed by the Jagiellonian University Medical College in Krakow. Anonymously collected data is deposited with the use of a dedicated online questionnaire. Data from a network of 161 invasive cardiology facilities in Poland between January 2014 to December 2021 were evaluated. On 4 March 2020 the first case of confirmed COVID-19 in Poland was announced. For this study, this day was classified as the beginning of the COVID-19 pandemic. Patients’ allocation and the scheme of the flow chart are shown in Figure 1.

During the COVID-19 outbreak, all patients with STEMI underwent swab tests for the qualitative detection of nucleic acid from SARS-CoV-2 in the ambulance or at the destination hospital. To avoid treatment delay, the outcome of a real-time reverse transcription-polymerase chain reaction test was not awaited. All patients were treated as suspected to have a positive result for COVID-19. All procedures were conducted ensuring local standards of PCI and recommendations from the European Society of Cardiology (ESC) [18]. All periprocedural complications and adverse outcomes were assembled prospectively by local physicians in compliance with descriptions in current ESC guidelines [18]. Neither detailed data on complexity, lesion type, nor follow-up after discharge from the hospital were available. Furthermore, there was lack of prehospital data, such as time of CPR by medical staff/bystanders or automated external defibrillator (AED) utilization. Out-of-hospital cardiac arrest was defined as the sudden cessation of the organized electrical activity of cardiomyocytes with the loss of mechanical contractility and ability to supply effective blood circulation diagnosed outside the hospital. Definitions of all complications related to PCI were described previously [3,12,13,14,15,16,19]. Briefly, periprocedural death was expressed as all-cause mortality from PCI procedure onset until transfer to the cardiology department or the intensive care unit. Bleeding complications were determined according to definitions from the Bleeding Academic Research Consortium [20]. Cerebrovascular complications were established on the base of the diagnosis of local physicians and clinical presentation. Detailed data on the type of stroke and long-term neurological observation were not collected. The institutional ethical board affirmed the study. All included patients dispensed, personally signed informed consent forms for the procedure. The study was provided in congruence with ethical principles derived from the Declaration of Helsinki with later amendments. No financial support was provided for this registry.

### Statistical Methods

A propensity score matching was conducted to imitate the randomization process and avoid potential preselection bias associated with the study design. A multivariate logistic regression model was calculated with the time of OHCA (before versus during the COVID-19 pandemic) set as the dependent variable. All baseline characteristics (gender, age, weight, diabetes mellitus, previous stroke, previous myocardial infarction, previous PCI, previous coronary artery bypass grafting (CABG), chronic obstructive pulmonary disease, smoking status, arterial hypertension, chronic kidney disease, psoriasis, Killip-Kimball class on admission, periprocedural treatment: angiography results, vascular access site, aspiration thrombectomy, rotablation, contrast volume, radiation dose, acetylsalicylic acid, P2Y_12_ inhibitors, unfractionated heparin, low-molecular-weight heparin, glycoprotein IIb/IIIa inhibitors, bivalirudin; baseline clinical data: thrombolysis in myocardial infarction (TIMI) scale before PCI, operator experience using radial access site in PCI procedures, direct transport, site volume ≥400 PCIs) were set as covariates. The nearest neighbor approach was utilized to attain an acceptable balance resulting in standardized differences for all confounders determined as below 10%. Patients were matched in a 1:1 scheme. Unpaired patients were excluded from matched-paired evaluation. Typical descriptive statistics were calculated in the analysis. Quantitative variables were expressed as mean and standard deviation. Categorical variables were described as counts and percentages. The Mann–Whitney U test (for non-normally distributed data) or Student’s *t*-test (for normally distributed data) for continuous variables and Fisher’s exact test or Pearson’s chi-squared test for categorical (nominal and dichotomous) variables were utilized to assess the intragroup differences. The normality of the data distribution was evaluated with the Shapiro–Wilk test for the sample size below 2000, and the Kolmogorov–Smirnov test with Lilliefors correction was calculated for samples over 2000. Matched pairs of subjects were compared with the Wilcoxon-signed-rank test (for non-normally distributed data difference) or the paired *t*-test (for normally distributed data difference) for continuous variables and the McNemar–Bowker’s test for categorical (nominal) variables. Two-sided *p*-values <0.05 were reckoned as statistically significant. To account for the loss of observations, which is a natural result of using propensity score matching, we validated the propensity score results using multiple regression analysis, including the variable of interest, as well as all variables from the original propensity score model. Backward selection in logistic regression analysis with a probability value for covariates to enter the model was set at 0.05. Results were presented as odds ratios (OR) with 95% confidence intervals (CI). All statistical calculations were conducted with JMP^®^, Version 16.2.0 (SAS Institute Inc., Cary, NC, USA, 2020) and R version 4.1.1 (R Core Team, Vienna, Austria, 2021) with package MatchIt 4.2.0.

## 3. Results

**Figure 2:**
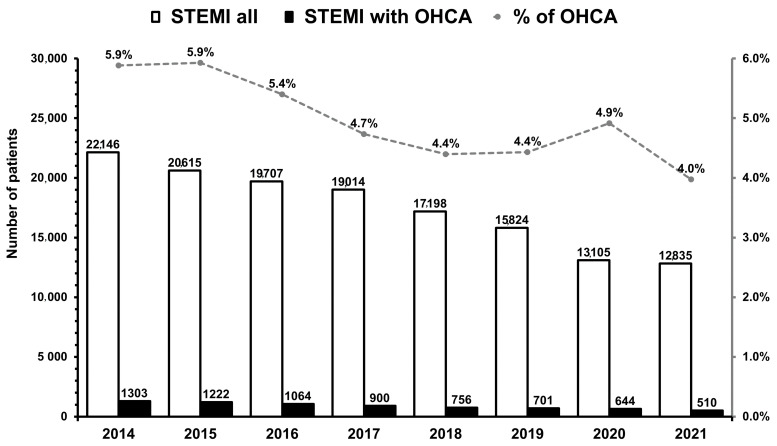
The total number of patients with STEMI and STEMI complicated by OHCA between 2014 and 2021 (*p* = 0.001). The dotted line represents the proportion of STEMI with OHCA to STEMI in each year.; STEMI—ST-segment-elevation myocardial, OHCA—out-of-hospital cardiac arrest.

There was a substantial reduction in the number of STEMI and STEMI complicated by OHCA during the COVID-19 outbreak compared to the pre-pandemic era (Figure 2). 

However, a higher proportion of STEMI with OHCA to STEMI was observed during the peak of the COVID-19 pandemic as compared to previous years. Overall, 5501 consecutive patients with STEMI complicated by OHCA and treated with primary PCI with stent implantation were enrolled. Fibrinolytic therapy was administered to none of the included patients. Complete baseline clinical and demographic characteristics are presented in Table 1. Data before propensity score matching are gathered in supplementary Tables S1–S5.

A total of 740 matched pairs of patients with STEMI and OHCA treated before and during the COVID-19 pandemic were compared. Both groups were well matched, with no differences in baseline presentation (Table 1). All demonstrated data were computed for matched pairs. A comparable range of coronary artery disease severity in angiography, as well as TIMI flow grades before and after PCI, were detected in both groups (Table 2). 

Furthermore, there was no difference in antiplatelet and antithrombotic therapy. Similarly, invasive cardiologists with equal dexterity and experience performed procedures in both groups. Additionally, radiation doses and the total amount of contrast were comparable before and during the COVID-19 pandemic (Table 3). 

Patients treated during the COVID-19 outbreak experienced longer delays from first medical contact to angiography (88.8 (±61.5) vs. 101.4 (±109.8) [minutes]; *p* = 0.006). There was also a trend toward prolonged time from pain onset to angiography in patients admitted to the hospital in the pandemic era (207.3 (±192.8) vs. 227.9 (±231.4) [minutes]; *p* = 0.06). Conversely, the time from chest pain onset to first medical contact was similar in both groups (Table 4).

A similar periprocedural mortality and the prevalence of any periprocedural complications were observed despite the enrollment period (Table 5). Data from multivariate regression analysis confirmed no increase in the risk of periprocedural death for the COVID-19 pandemic period (OR 0.7, 95% CI 0.44–1.11; *p* = 0.1)

## 4. Discussion

The results of this study suggested no impact of the COVID-19 pandemic on periprocedural outcomes in patients with STEMI complicated by OHCA. Similar treatment patterns were observed both before and during the outbreak. However, patients treated during the COVID-19 era were exposed to a longer time to reperfusion. To the best of our knowledge, this is the first study to evaluate clinical outcomes in STEMI complicated by OHCA during the COVID-19 pandemic as compared to pre-pandemic era. A decrease in the number of STEMI patients and a higher rate of OHCA counts were widely reported despite geographical location since the pandemic began [1,2,3,4,7,21,22,23,24,25,26]. Several factors might partially explain these findings. Deterioration of access to healthcare and reluctance to seek medical care in STEMI symptoms might lead to a more severe condition manifested with OHCA and prehospital death [2,7,9,21,27]. Interestingly, the North American COVID-19 Myocardial Infarction registry reported more frequently atypical symptoms of STEMI with a potentially deceptive impact on patients [21,27]. Overcrowded hospitals with mandatory swab tests and pandemic-specific protocols might also prolong emergency health services’ response time. Altogether, these factors might prolong the time to PCI. Notably, previous reports demonstrated a reduced survival with every minute of delay to revascularization [21,22,23,24,25,26,27,28]. The continuous relationship between shorter delay in PCI and reduced mortality suggests no specific threshold time for acceptable treatment defer [21,22,23,24,25,26,27,28]. Thus, another convincing explanation for decreasing the number of STEMI might be a higher incidence of fatal sudden cardiac arrest before hospital admission compared to the pre-pandemic era. Importantly, such disturbing outcomes were noted irrespective of the geographical location and in low- and high-income countries with well-developed healthcare systems [7,9,26,29,30,31]. Furthermore, alarming data suggested a decrease in bystander CPR, utilization of AED, and a higher rate of non-shockable rhythms [1,7,9]. Evaluation of CPR practices after the onset of the COVID-19 pandemic highlighted a decrease in the willingness to perform life-saving interventions despite SARS-CoV-2 status [7,9,24,25,26]. Fear of COVID-19 exposure might explain declined readiness to render life-saving maneuvers. This phenomenon might be associated with a higher rate of mortality before PCI and hospital admission. Thus, some patients with the highest burden of risk might not be incorporated in this study. All these factors might fractionally contribute to the suggested change in the risk of prehospital mortality and rate of OHCA in STEMI. Our findings are consistent with the alarming data highlighted by the aforementioned studies. A longer time from first medical contact to angiography was observed in patients with STEMI with OHCA treated during the COVID-19 pandemic. However, it did not result in difference in periprocedural mortality and other complications between the pandemic and pre-pandemic period. Unfortunately, only periprocedural data were collected in our study, which may not reflect the overall risk of included patients. Nevertheless, patients with STEMI and OHCA are at extremely high risk of mortality in spite of the pandemic or pre-pandemic period. On the contrary, detrimental effects on in-hospital survival, increased OHCA incidence, and generally worse outcomes were confirmed in a few meta-analyses [7,24,26]. In addition, despite consistency among available data, there is potential bias related to different populations, organization of healthcare systems, treatment strategy, and endpoint definitions. Meta-analyses included mostly retrospective observational studies with significant heterogeneity between participating centers. Thus, the generalizability of the results and direct comparison with more homogenous and prospectively collected data is limited. Furthermore, large meta-analyses also included data from an early phase of the pandemic. Worldwide exponential progress in evidence and experience might provide more optimistic outcomes from contemporary data. Notably, only a relatively short follow-up is available up to date. Thus, a further accumulation of data might allow for sufficient analysis. Despite the detrimental global outcome of OHCA during the COVID-19 era, the previously published meta-analysis demonstrated an increased risk of short-term mortality in STEMI patients treated in the COVID-19 pandemic compared to previous years [32]. However, this disturbing outcome was reported only in low/middle-income countries. Thus, there should be more concern about healthcare systems ineffectively struggling with STEMI patients during the COVID-19 outbreak. All endeavor is crucial to reduce treatment delay in STEMI patients, especially during the COVID-19 pandemic. Social campaigns should provide education and encourage patients to seek medical care in the early phase of symptoms to achieve timely management. Furthermore, the promotion of CPR education among communities is essential to retain a robust response to OHCA and avoid depraving collapse in bystander CPR [23,24,25,26,33].

### Limitations

The crucial drawback of this study is the non-randomized pattern with all-acquired bias. The endangerment of an unmeasured confounding coefficient cannot be ruled out. However, a propensity score match analysis was conducted to overcome this limitation. Furthermore, the presented study might not encompass all STEMI populations during the study’s extent. Patients with the highest burden of risk might die before admission to the invasive cardiology center. Another major limitation is a lack of prehospital data, including the time of cardiac arrest, initiation of CPR by a random witness of OHCA, or use of AED. Experience and personal skills in CPR were also not incorporated in the analysis; thus, it might also be an important limitation. Furthermore, follow-up data beyond catheterization laboratory were not collected. No further neurological assessment was available. The influence of the duration and variant of mechanical ventilation or circulatory support was not involved in the database. Long-term observation might be essential for a complete evaluation of the impact of the COVID-19 pandemic on clinical outcomes in this group of patients. Finally, the sample size might not be sufficient to detect the difference in mortality and adverse event rates. Regardless of all these limitations, our study exemplified national practice from a large unselected cohort of patients. Thus, we revealed comprehensive insights into real-world clinical practice in STEMI patients complicated by OHCA before and during the COVID-19 outbreak. 

## 5. Conclusions

Patients with STEMI complicated by OHCA treated during the COVID-19 outbreak experienced a longer time from the first medical contact to revascularization. However, the periprocedural outcomes of those patients were comparable before and during the COVID-19 era. Public education and promoting life-saving interventions undertaken by bystanders might be crucial to minimize total myocardial ischemia time and retain a robust response to OHCA during the COVID-19 pandemic.

## Figures and Tables

**Table 1:** Baseline characteristics after propensity score matching.

Variable	Before COVID-19 (n = 740)	During COVID-19 (n = 740)	*p*-Value
Male gender	512 (69.2%)	525 (70.9%)	0.5
Age [years]	63.8 (±12.4)	64.3 (±12.2)	0.6
Diabetes mellitus	111 (15%)	132 (17.8%)	0.2
Previous stroke	36 (4.9%)	29 (3.9%)	0.5
Previous MI	93 (12.6%)	96 (13%)	0.3
Previous CABG	12 (1.6%)	14 (1.9%)	0.8
Previous PCI	83 (11.2%)	87 (11.8%)	0.8
Smoking	238 (32.2%)	220 (29.7%)	0.4
Arterial hypertension	377 (50.9%)	385 (52%)	0.8
Chronic kidney disease	27 (3.6%)	32 (4.3%)	0.6
Chronic obstructive Pulmonary disease	21 (2.8%)	19 (2.6%)	0.9
Killip-Kimball class			
I	323 (43.6%)	304 (41.1%)	0.7
II	133 (18%)	134 (18.1%)	0.7
III	54 (7.3%)	63 (8.5%)	0.7
IV	230 (31.1%)	239 (32.3%)	0.7
Direct transport	316 (42.7%)	297 (40.1%)	0.3

Data are presented as number (percentage) or mean and standard deviation. CABG—coronary artery bypass grafting; MI—myocardial infarction; PCI—percutaneous coronary intervention.

**Table 2:** Angiographic characteristics after propensity score matching.

Variable	Before COVID-19 (n = 740)	During COVID-19 (n = 740)	*p*-Value
Single-vessel disease	325 (43.9%)	317 (42.8%)	0.8
LMCA only	5 (0.7%)	5 (0.7%)	0.8
Multivessel disease without LMCA	328 (44.3%)	345 (46.6%)	0.8
Multivessel disease with LMCA	82 (11.1%)	73 (9.9%)	0.8
TIMI 0 or 1 flow before PCI	599 (80.9%)	590 (79.7%)	0.8
TIMI 3 flow after PCI	623 (84.2%)	626 (84.6%)	0.9

Data are presented as numbers (percentage). LMCA—left main coronary artery; PCI—percutaneous coronary intervention; TIMI—thrombolysis in myocardial infarction.

**Table 3:** Percutaneous coronary intervention details after propensity match score.

Variable	Before COVID-19 (n = 740)	During COVID-19 (n = 740)	*p*-Value
Site volume ≥400 PCI in the current year	701 (94.7%)	692 (93.5%)	0.4
Radial approach during angiography	420 (56.8%)	436 (58.9%)	0.6
Radial approach during PCI	412 (55.7%)	428 (57.8%)	0.7
PCI operator annual volume (PCI during 2014–2021)	172.9 (±94.3)	170.7 (±103.6)	0.8
PCI operator radial experience (2014–2021) [% of all performed PCI]	82 (±14.6)	82.6 (±13.7)	0.4
Total amount of contrast, [ml]	158 (±62)	158 (±66)	0.9
Total radiation dose, [mGy]	750 (±590)	740 (±740)	0.8
Aspiration thrombectomy during PCI	113 (15.3%)	107 (14.5%)	0.7
Rotablation during PCI	3 (0.4%)	5 (0.4%)	0.9
P2Y12 inhibitors before and during PCI			
Clopidogrel	455 (61.5%)	459 (62%)	0.9
Ticagrelor	273 (36.9%)	270 (36.5%)	0.9
Prasugrel	12 (1.6%)	11 (1.5%)	0.9
GPI IIb/IIIa during PCI	225 (30.4%)	237 (32%)	0.5
Unfractionated heparin during PCI	636 (85.9%)	630 (85.1%)	0.7
Low-molecular-weight heparins during PCI	6 (0.8%)	8 (1.1%)	0.8
Bivalirudin during PCI	8 (1.1%)	7 (0.9%)	0.9

Data are presented as numbers (percentage) or mean and standard deviation. PCI—percutaneous coronary intervention.

**Table 4:** Treatment delays after propensity score matching.

Variable	Before COVID-19 (n = 740)	During COVID-19 (n = 740)	*p*-Value
Time from pain to first medical contact, minutes	118.5 (±173.6)	126.5 (±197.5)	0.4
Time from pain to angiography, minutes	207.3 (±192.8)	227.9 (±231.4)	0.06
Time from first medical contact to angiography, minutes	88.8 (±61.5)	101.4 (±109.8)	0.006
Time from first medical contact to angiography <90 minutes, %	286 (38.6%)	283 (38.2%)	0.9
Time from first medical contact to angiography <120 minutes, %	161 (21.8%)	168 (22.7%)	0.7

Data are presented as numbers (percentage) or mean and standard deviation.

**Table 5:** Periprocedural complications after propensity score matching.

Variable	Before COVID-19 (n = 740)	During COVID-19 (n = 740)	*p*-Value
Dissection of coronary artery	0 (0.0%)	1 (0.1%)	0.9
Coronary artery perforation	3 (0.4%)	1 (0.1%)	0.6
No-reflow	20 (2.7%)	19 (2.6%)	0.9
Periprocedural stroke	0	0	-
Bleeding at the puncture site	2 (0.3%)	2 (0.3%)	0.9
Allergic reaction	0	0	-
Cardiac arrest	64 (8.6%)	47 (6.4%)	0.1
Periprocedural death	45 (6.1%)	32 (4.3%)	0.2
Periprocedural myocardial infarction	0	0	-
Any complication	103 (13.9%)	82 (11.1%)	0.1
Any complication or death	91 (12.3%)	73 (9.9%)	0.2

Data are presented as numbers (percentage).

## Supplementary Materials

The following supporting information can be downloaded at: https://www.mdpi.com/article/10.3390/ijerph20010337/s1, Table S1: Baseline characteristics before propensity score matching; Table S2: Angiographic characteristics before propensity score matching; Table S3: Percutaneous coronary intervention details before propensity match score; Table S4: Treatment delays before propensity score matching; Table S5: Periprocedural complications before propensity score matching.
