# Supplementary material for: Impact of COVID-19 Pandemic on Patients with ST-Segment-Elevation Myocardial Infarction Complicated by Out-of-Hospital Cardiac Arrest
Source: Int J Environ Res Public Health. 2022 Dec 26;20(1):337. doi: 10.3390/ijerph20010337 (PMC9819125; doi:10.3390/ijerph20010337)
Supplement: Supplementary file 1 [file ijerph-20-00337-s001.zip › ijerph-2078255-Supplementary Tables.pdf]

Supplementary Table S1. Baseline characteristics before propensity score matching.

Variable	Before COVID-19 (n = 4671)	During COVID-19 (n = 740)	p-value
Male gender	3300 (70.6%)	525 (70.9%)	0.9
Age [years]	64.7 (±12.2)	64.3 (±12.2)	0.5
Diabetes mellitus	747 (16%)	132 (17.8%)	0.02
Previous stroke	210 (4.5%)	29 (3.9%)	0.5
Previous MI	679 (14.5%)	96 (13%)	0.03
Previous CABG	82 (1.8%)	14 (1.9%)	0.8
Previous PCI	559 (12%)	87 (11.8%)	0.8
Smoking	1202 (25.7%)	220 (29.7%)	0.02
Arterial hypertension	2416 (51.7%)	385 (52.0%)	0.9
Chronic kidney disease	218 (4.7%)	32 (4.3%)	0.8
Chronic obstructive pulmonary disease	80 (1.7%)	19 (2.6%)	0.1
Killip-Kimball Class			
I	2187 (46.8%)	304 (41.1%)	0.008
II	693 (14.8%)	134 (18.1%)	0.008
III	434 (9.3%)	63 (8.5%)	0.008
IV	1357 (29.1%)	239 (32.3%)	0.008
Direct transport	1640 (35.1%)	297 (40.1%)	0.009

Data are presented as number (percentage) or mean and standard deviation. CABG - coronary artery bypass grafting; MI - myocardial infarction; PCI - percutaneous coronary intervention.

Supplementary Table S2. Angiographic characteristics before propensity score matching

Variable	Before COVID-19 (n = 4671)	During COVID-19 (n = 740)	p-value
Single-vessel disease	1894 (40.5%)	317 (42.8%)	0.1
LMCA only	46 (1.0)	5 (0.7)	0.5
Multivessel disease without LMCA	2228 (47.7)	345 (46.6)	0.5
Multivessel disease with LMCA	503 (10.8)	73 (9.9)	0.8
TIMI 0 or 1 flow before PCI	3673 (78.6)	590 (79.7)	0.5
TIMI 3 flow after PCI	4020 (86.1)	626 (84.6)	0.1

Data are presented as number (percentage). LMCA - left main coronary artery; PCI - percutaneous coronary intervention; TIMI - thrombolysis in myocardial infarction

Supplementary Table S3. Percutaneous coronary intervention details before propensity match score

Variable	Before COVID-19 (n = 4671)	During COVID-19 (n = 740)	p-value
Site volume ≥400 PCI in current year	4340 (92.9)	692 (93.5)	0.6
Radial approach during angiography	2012 (43.1%)	436 (58.9%)	0.001
Radial approach during PCI	1989 (42.6%)	428 (57.8%)	0.001
PCI operator annual volume (PCI during 2014-2021)	180.3 (±113.9)	170.7 (±103.6)	0.03
PCI operator radial experience (2014-2021) [% of all performed PCI]	78.0 (±19.1)	82.6 (±13.7)	0.001
Total amount of contrast, [ml]	174 (±75)	158 (±66)	0.001
Total radiation dose, [mGy]	1040 (±1020)	740 (±740)	0.001
Aspiration thrombectomy during PCI	779 (16.7%)	107 (14.5%)	0.04
Rotablation during PCI	4 (0.1)	5 (0.7)	0.01
P2Y12 inhibitors before and during PCI			
Clopidogrel	3514 (75.2%)	459 (62%)	0.001
Ticagrelor	1112 (23.8%)	270 (36.5%)	0.001
Prasugrel	45 (1.0%)	11 (1.5%)	0.001
GPI IIb/IIIa during PCI	1257 (26.9)	237 (32.0)	0.001
Unfractionated heparin during PCI	3925 (84.0)	630 (85.1)	0.7

Low-molecular-weight heparins during PCI	122 (2.6)	8 (1.1)	0.02
Bivalirudin during PCI	17 (0.4)	7 (0.9)	0.05

Data are presented as number (percentage) or mean and standard deviation. PCI - percutaneous coronary intervention

Supplementary Table S4. Treatment delays before propensity score matching.

Variable	Before COVID-19 (n = 4671)	During COVID-19 (n = 740)	p-value
Time from pain to first medical contact, minutes	122.1 (±180.8)	126.5 (±197.5)	0.5
Time from pain to angiography, minutes	230.4 (±218.5)	227.9 (±231.4)	0.8
Time from first medical contact to angiography, minutes	108.2 (±116.4)	101.4 (±109.8)	0.1
Time from first medical contact to angiography <90 minutes, %	2108 (45.1%)	283 (38.2%)	0.001
Time from first medical contact to angiography <120 minutes, %	1289 (27.6%)	168 (22.7%)	0.006

Data are presented as number (percentage) or mean and standard deviation

Supplementary Table S5. Periprocedural complications before propensity score matching.

Variable	Before COVID-19 (n = 4671)	During COVID-19 (n = 740)	P-value
Dissection of coronary artery	9 (0.2%)	1 (0.1%)	0.9
Coronary artery perforation	13 (0.3%)	1 (0.1%)	0.7
No-reflow	139 (3.0%)	19 (2.6%)	0.6
Periprocedural stroke	0	0	-
Bleeding at the puncture site	14 (0.3%)	2 (0.3%)	0.9
Allergic reaction	3 (0.05%)	0 (0%)	0.1
Cardiac arrest	358 (7.7%)	47 (6.4%)	0.2
Periprocedural death	313 (6.7%)	32 (4.3%)	0.02
Periprocedural myocardial infarction	1 (0.01%)	0 (0%)	0.9
Any complication	663 (14.2%)	82 (11.1%)	0.03
Any complication or death	559 (12.0%)	73 (9.9%)	0.1

Data are presented as number (percentage).
